# A Manual of Treatment by Massage

**Published:** 1887-08

**Authors:** 


A MANUAL OF TREATMENT BY MASSAGE AND METHODICAL MUSCLE EXERCISE. By Joseph Schreiber, M. D., Member of the K. K. Geseleschaft der Aertze of Vienna, etc. Translated by Walter Mendelson, M. D., of New York. Philadelphia: Lea Brothers & Co. 1887.

The history of massage as a mode of treatment of disease is quite similar to that of the medical use of electricity. Possessing just enough of scientific basis to give to it an air of plausibility and to secure for it, in a limited number of cases, successful results, it unfortunately fell into the hands of empirics and charlatans and was for a long time used to play upon the ignorance and credulity of the laity. For this reason it was shunned by the more respectable portion of the profession as a species of quackery, until rescued from this unmerited obloquy by the systematic and scientific investigations of a few enlightened observers. The results of such investigations are embodied in the volume before us, and so impartially are the claims of this mode of treatment considered and so clearly are its advantages set forth that no intelligent reader will be disposed to deny that there are good grounds for the views therein advanced. That the author is evidently an enthusiast does not detract from the value of his work, since none but an enthusiast is capable of properly presenting a new idea to so conservative a body as the medical profession. As Herbert Spencer has said, "It is only by varied iteration that an alien conception can be forced upon reluctant minds." As a thorough and satisfactory exposition of the science of mechanical therapeutics, adapted to the use of the general practitioner, this volumes leaves nothing to be desired. The text is fully illustrated by well-drawn wood-cuts, leaving no room for obscurity in the description of the various manipulations recommended. The book does credit to the author, the translator and the publishers.

